# Comparison of Different Machine Models Based on Multi-Phase Computed Tomography Radiomic Analysis to Differentiate Parotid Basal Cell Adenoma From Pleomorphic Adenoma

**DOI:** 10.3389/fonc.2022.889833

**Published:** 2022-07-12

**Authors:** Yun-lin Zheng, Yi-neng Zheng, Chuan-fei Li, Jue-ni Gao, Xin-yu Zhang, Xin-yi Li, Di Zhou, Ming Wen

**Affiliations:** ^1^ Department of Radiology, The First Affiliated Hospital of Chongqing Medical University, Chongqing, China; ^2^ Department of Gastroenterology, The Second Affiliated Hospital, Chongqing Medical University, Chongqing, China

**Keywords:** radiomic analysis, machine learning, computed tomography, parotid pleomorphic adenoma, basal cell adenoma

## Abstract

**Objective:**

This study explored the value of different radiomic models based on multiphase computed tomography in differentiating parotid pleomorphic adenoma (PA) and basal cell tumor (BCA) concerning the predominant phase and the optimal radiomic model.

**Methods:**

This study enrolled 173 patients with pathologically confirmed parotid tumors (training cohort: n=121; testing cohort: n=52). Radiomic features were extracted from the nonenhanced, arterial, venous, and delayed phases CT images. After dimensionality reduction and screening, logistic regression (LR), K-nearest neighbor (KNN) and support vector machine (SVM) were applied to develop radiomic models. The optimal radiomic model was selected by using ROC curve analysis. Univariate and multivariable logistic regression was performed to analyze clinical-radiological characteristics and to identify variables for developing a clinical model. A combined model was constructed by integrating clinical and radiomic features. Model performances were assessed by ROC curve analysis.

**Results:**

A total of 1036 radiomic features were extracted from each phase of CT images. Sixteen radiomic features were considered valuable by dimensionality reduction and screening. Among radiomic models, the SVM model of the arterial and delayed phases showed superior predictive efficiency and robustness (AUC, training cohort: 0.822, 0.838; testing cohort: 0.752, 0.751). The discriminatory capability of the combined model was the best (AUC, training cohort: 0.885; testing cohort: 0.834).

**Conclusions:**

The diagnostic performance of the arterial and delayed phases contributed more than other phases. However, the combined model demonstrated excellent ability to distinguish BCA from PA, which may provide a non-invasive and efficient method for clinical decision-making.

## Introduction

Parotid gland tumors are the predominant salivary gland tumors, more than 80% of which are benign (1). According to the 5th Edition of the World Health Organization (WHO) classification of Head and Neck Tumors, there were 15 histological types of benign parotid tumors, of which the most common type was pleomorphic adenoma (PA), followed by Warthin tumor (WT) and basal cell adenoma (BCA) (2, 3). Most patients with benign parotid tumors present as a painless mass, frequently asymptomatic and incidentally detected (4). While BCA is a rare benign tumor, the incidence rises each year (5). Due to physicians’ lack of a more detailed awareness, it is commonly mistaken for a similar PA. Although both are benign tumors, the biology and surgical procedures are entirely different, such as PA has a higher recurrence and malignancy rate and a more comprehensive surgical range than BCA (6–8). To optimize the individualized surgical approach, reduce the incidence of postoperative complications, and inform preoperative patient counseling it is essential to distinguish preoperatively between PA and BCA (8, 9).

Fine needle aspiration biopsy (FNAB) is commonly utilized in the preoperative qualitative diagnosis of parotid tumors, but its effectiveness is controversial (10, 11). Therefore, preoperative imaging, especially CT and MRI, has a crucial role in assessing the location, nature, and relationship to surrounding structures of parotid tumors (12, 13). While MRI has high contrast soft-tissue resolution, its drawbacks are high cost, prolonged image acquisition time, and susceptibility to motion artifacts (14). In contrast, multiphase enhanced CT has the advantages of being convenient to operate, cheaper, and widely available for identifying parotid tumors (6, 15). The radiological features of BCA and PA overlap, thus accurately differentiating the two remains a challenge for most physicians, and their diagnostic accuracy depends strongly on observer expertise and experience (16).

Radiomics, an emerging and rapidly developing field, integrates radiology, oncology, and machine learning to perform an essential role in precision diagnosis, tumor treatment, and prognosis through high-throughput extraction and mining of a large number of image features (17). In recent years, with the advancement of artificial intelligence, the application of radiomics to identify parotid tumors has made significant progress (18–20). The high accuracy, effectiveness, and reliability of prediction models are critical factors in facilitating the success of radiomics; thus, it is essential to compare different machine learning models based on radiomics (21). To our knowledge, nevertheless, no studies have investigated the different radiomic models applied to distinguish BCA from PA.

This study first investigated the performance of different radiomic models and the dominant scan phase from multiphasic CT in differentiating BCA from PA. In particular, clinical-radiological features and radiomics were combined to differentiate BCA from PA.

## Materials and Methods

### The Patient Cohort and CT Image Acquisition

**Figure 1:**
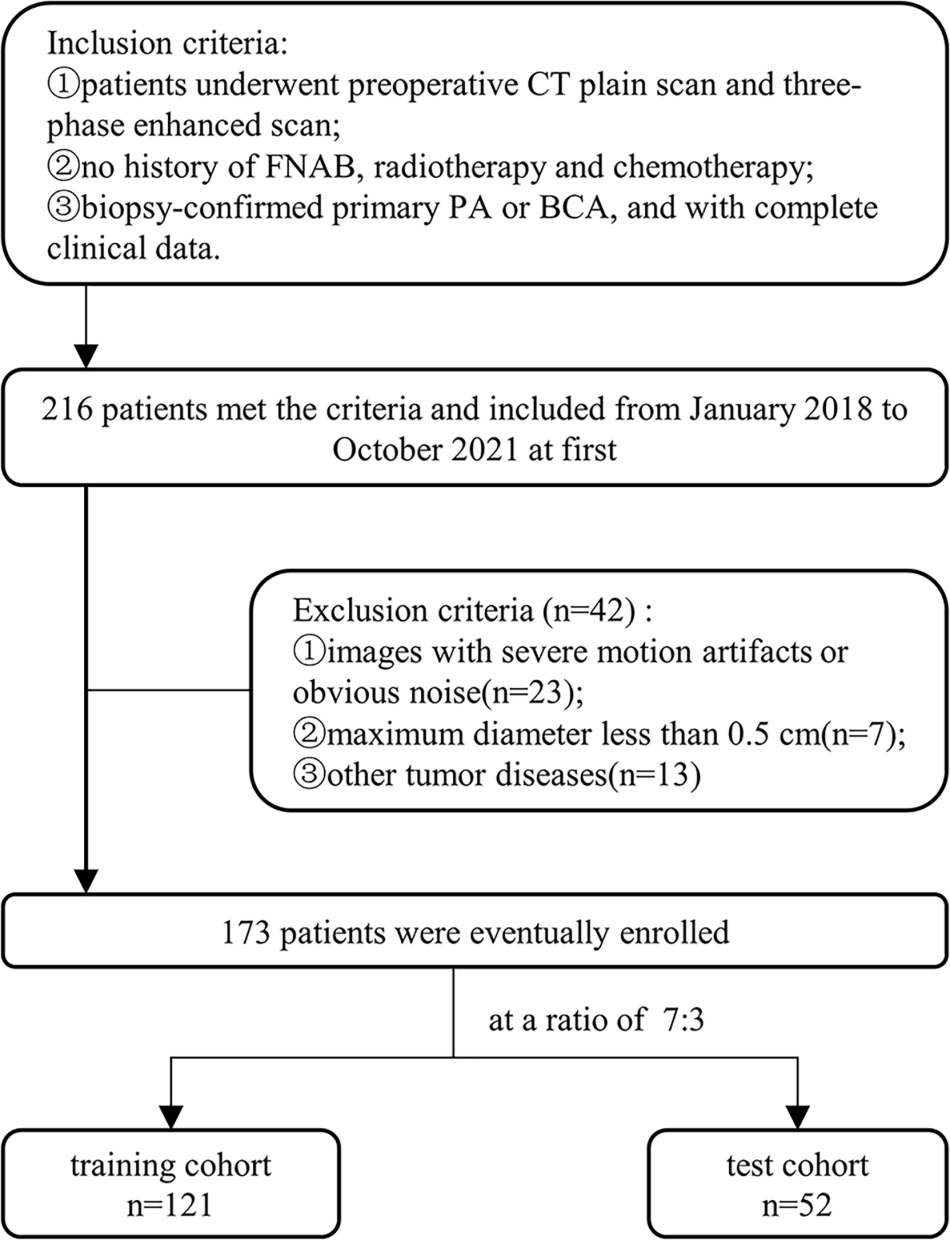
Workflow for patient enrollment.

This retrospective analysis included eligible patients in our hospital from January 2018 to October 2021, was approved by the Ethics Review Committee of our institution (approval number 2022-K34), and exempted from informed consent requirements. In this study, the patient selection and exclusion criteria were illustrated in **Figure 1**. Overall, 173 eligible patients were enrolled, including 121 cases of PA (50 males and 71 females; age 45.3 ± 15.9 years) and 52 cases of BCA (20 males and 32 females; age 54.4 ± 11.8 years). These patients were randomized in a 7:3 ratio into a training and testing cohort.

CT scans were conducted with two 64 multidetector CT scanners (Discovery 750, GE Healthcare, Milwaukee, WI, USA; Somatom Sensation 64, Siemens Healthcare, Erlangen, Germany) with the following parameters: 120 kV; tube current modulation (150–240mAs); matrix, 512 × 512; section thickness, 5 mm; section interval, 5 mm. Non-ionic iodinated contrast medium (Ultravist 350 mg/ml, Bayer Schering Pharma, Berlin, Germany) was injected into the anterior cubital vein (dose 1.5 ml/kg) at a flow rate of 3.5 ml/s for all contrast-enhanced scans. All images were acquired from the skull base to the thorax inlet. Multiphase scans were collected for each patient: non-enhanced scan, arterial phase scan (40 seconds after intravenous contrast injection), venous phase scan (after 100 seconds), and delayed phase scan (after 4 or 5 minutes).

### Image Segmentation, Radiomic Features Extraction and Selection

Multiphase CT images were stored in Digital Imaging and Communications in Medicine (DICOM) format and imported into ITK-SNAP software (version 3.8.0; http://www.itksnap.org/) for three-dimensional manual segmentation of regions of interest (ROIs). The ROIs were sketched along boundaries layer-by-layer on axial images by a radiologist with 5 years of experience and reviewed by another radiologist with 12 years of experience, blinded to the clinical information.

To extract robust features and assure model reproducibility, intra-observer intraclass correlation coefficients (ICC) were assessed. Fifty-two patients (36 PA and 16 BCA) were randomly selected for ROI segmentation again 1 month later by the same radiologist, and an ICC greater than 0.75 suggested good consistency (20). Image preprocessing was performed on the images and ROIs to counteract the potential effects of different imaging parameters on the extracted features, including resampling, normalizing, and discretizing the gray level of images (18). The package py-radiomics 3.0 in python software (version 3.7.6; https://www.python.org/) was employed for feature extraction. For each phase of CT images, 1036 radiomic features were extracted, including the shape, histogram, gray-level co-occurrence matrix (GLCM), gray-level dependence matrix (GLDM), gray-level run-length matrix (GLRLM), gray-level size zone matrix (GLSZM), and filter features (Laplacian of Gaussian and wavelet filters).

**Figure 2:**
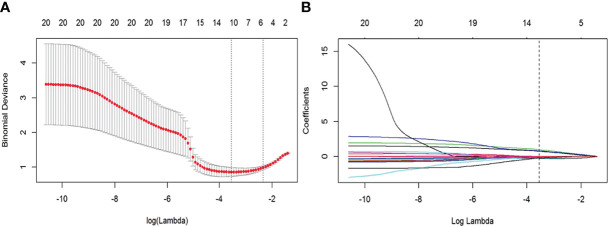
Feature selection with the least absolute shrinkage and selection operator (LASSO) regression method. **(A)** Variation of tuning parameter (λ) for LASSO model, using 10-fold cross-validation. The vertical dotted lines indicate the best λ=0.028 was selected with a least binomial deviation. **(B)** The LASSO coefficient profiles with radiomic features of different log (λ). The vertical dotted line was the best λ with 10 radiomic features with non-zero coefficients.

The feature dimensionality reduction and selection in the training cohort were as follows: first, the student’s t-test was used to select features that differed significantly between groups. Second, correlation analysis was applied to eliminate redundant parameters from advancement to model construction. If there was a high correlation (>0.8), the parameter with the high area under the receiver operating curve (ROC) was selected. Third, a least absolute shrinkage and selection operator (LASSO) regression model with 10-fold cross-validation was performed to select radiomic features with nonzero coefficients (**Figure 2**). All feature selection processes were performed on the training cohort and executed on the testing cohort. The final selected features were utilized for modeling.

### Different Radiomic Models Developing and Validation

In machine learning, classifiers were considered supervised learning tasks that deduced functions from training data and analyzed the training data to predict unobserved groups (21). To select a classifier model with the higher capability to identify tumor data, our study chose three mainstream classifier models, including logistic regression (LR), K-nearest neighbors (KNN), and support vector machine (SVM) (22). The diagnostic performance of the three models was estimated by the area under the curve (AUC) of the receiver operating characteristic (ROC) curve, accuracy, sensitivity, and specificity. Then, the dominant phase and radiomic model were screened based on our study data.

### Clinical and Radiological Data Analysis

Patients’ images and clinical data were taken from our institution’s routine clinical records and picture archiving and communication systems (PACS). We retrospectively analyzed the clinical parameters of age, disease duration, symptoms, gender, smoking/drinking status, inpatient number, and postoperative biopsy results. All CT images were assessed and deliberated upon separately by two radiologists with 5 and 34 years of head and neck experience, who were blinded to clinical results. The following parameters were evaluated: maximum diameter, tumor number, distribution, shape, boundary, tumor location, density, calcification, cystic degeneration, enhanced peak phase, enhancement degree, enhanced uniformity, and lymph node enlargement. Some radiological features were explained in the **Supplementary Material**. If there were multiple lesions in the parotid gland, the largest biopsy-confirmed lesion was chosen for analysis.

### Clinical and Combined Models Construction and Validation

**Figure 3:**
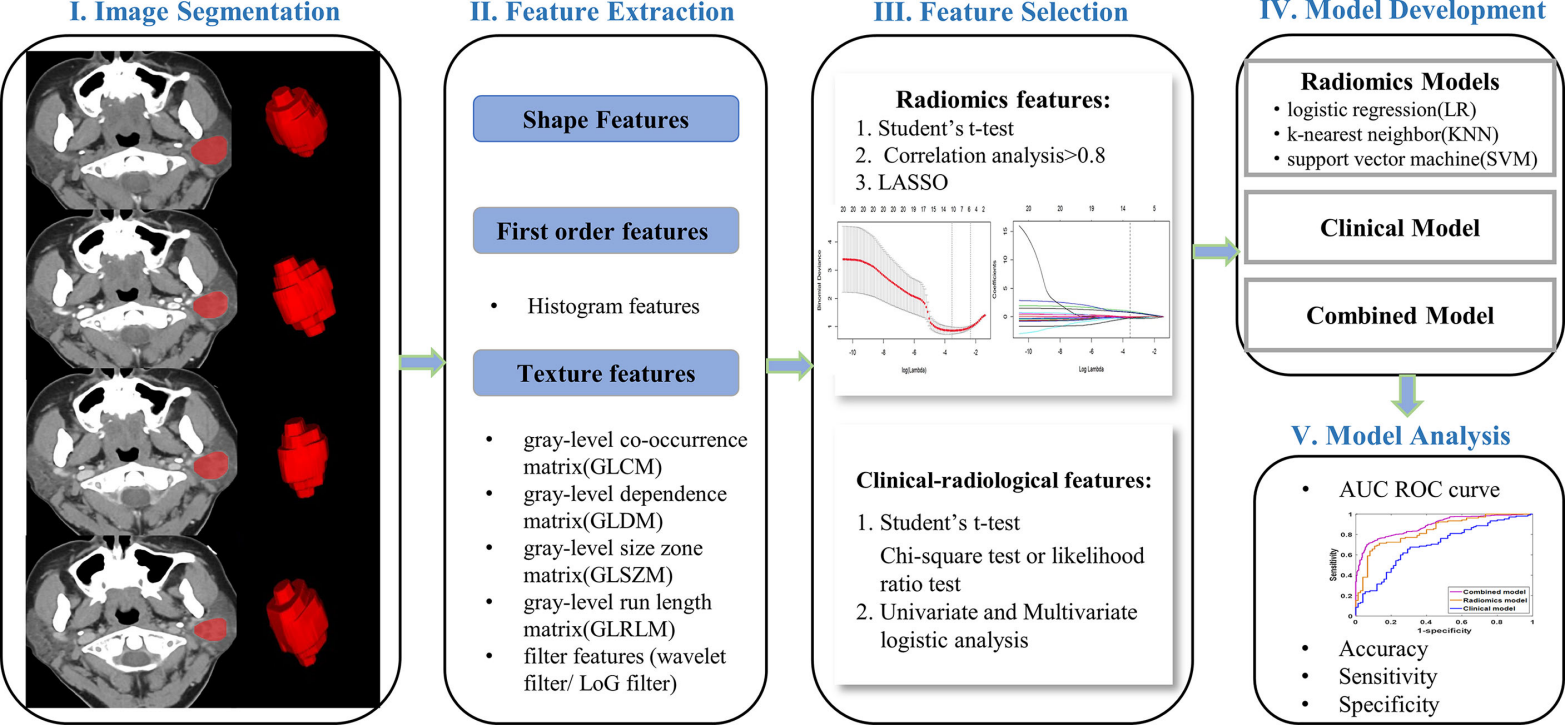
Flowchart of the present research protocol.

Univariate logistic regression analysis was conducted for each predictor variable in the training cohort, including clinical and radiological features, followed by multivariate logistic regression on statistically significant features to obtain final predictors for model development. Calculate the odds ratio (OR) and 95% confidence interval (CI) for each feature. We selected the optimal radiomic model to construct a combined model integrating clinical-radiological and four-phasic radiomic features for differentiating BCA from PA. The performances of different models were assessed and validated by training and testing cohorts, including AUC, accuracy, sensitivity, and specificity. A complete schematic was shown in **Figure 3**.

### Statistical Analysis

Statistical analysis was performed with SPSS 26.0 software (IBM, Chicago, IL, USA) and R software (version 3.6.3; https://www.r-project.org/). Normally distributed continuous variables were expressed as mean value ± standard deviation using the student t-test. Categorical variables, presented as ratios, were applied to the chi-square or likelihood ratio test. The LASSO, LR, KNN, and SVM models were conducted based on ‘glmnet’, ‘glmnet’, ‘kknn’, and ‘e1071’ for R software packages or sklearn package for python software, respectively (23). All models were fitted with the caret R package (24). Statistical comparisons of AUCs among different classifiers with the DeLong test were used. A two-tailed *p* < 0.05 considered statistical significance.

## Results

### Radiomic Features, Different Radiomic Models Construction and Validation

A total of 4144 radiomic features were extracted from the four-phase CT image radiomic features. To ensure extraction stability, we eliminated features with ICC values below 0.75 and retained 841, 893, 908, and 859 radiomic features in non-enhanced, arterial, venous, and delayed phases, respectively. Then, 527, 379, 475, and 391 features were removed by t-test and correlation analysis from each phase, respectively. LASSO was applied to the rest features, and16 features with non-zero coefficients were kept after feature dimensionality reduction and selection (see **Table 1** for details). These features were applied to construct three models of LR, KNN, and SVM in the training cohort. The performance of three models in distinguishing BCA from PA was evaluated in the testing cohort.

**Table 1:** Radiomic features selection results.

CT scanning phase	ID	Radiomic features name	
Non-enhanced phase	1	wavelet-LHH_ GLCM _ClusterShade	
	2	log-sigma-5-0-mm-3D_GLDM_LargeDependenceLowGrayLevelEmphasis
	3	wavelet-HHL_ GLSZM _SmallAreaLowGrayLevelEmphasis	
	4	wavelet-LLL_ first order _Mean	
Arterial phase	1	log-sigma-5-0-mm-3D_ GLRLM _HighGrayLevelRunEmphasis	
	2	wavelet-HLH_ GLCM _ClusterShade	
	3	wavelet-HHL_ first order _Median	
	4	log-sigma-5-0-mm-3D_ GLCM _Id	
Venous phase	1	original_ GLCM _Correlation	
	2	log-sigma-5-0-mm-3D_ GLSZM _SmallAreaLowGrayLevelEmphasis	
	3	log-sigma-5-0-mm-3D_ GLDM _DependenceVariance	
	4	wavelet-HLL_ GLCM _Autocorrelation	
Delayed phase	1	wavelet-HLL_ GLRLM _LongRunHighGrayLevelEmphasis	
	2	wavelet-LHL_ GLCM _JointAverage	
	3	log-sigma-5-0-mm-3D_ GLCM _Idmn	
	4	wavelet-HLL_ GLCM _Autocorrelation	

GLCM, gray-level co-occurrence matrix; GLDM, gray-level dependence matrix; GLSZM, gray-level size zone matrix; GLRLM, gray-level run length matrix.

**Figure 4:**
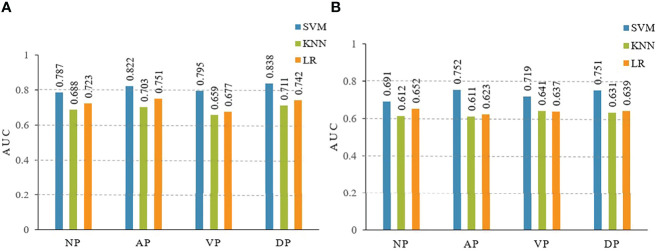
The area under the curves (AUCs) of receiver operating characteristics (ROC) curve analysis for three models of LR, KNN, SVM in the training **(A)** and testing **(B)** cohorts. LR, logistic regression; KNN, K-nearest neighbor; SVM, support vector machine. NP, non-enhanced phase; AP, arterial phase; VP, venous phase; DP, delayed phase.

The diagnostic performances of the three radiomic models (including LR, KNN, and SVM) are summarized in **Table 2**. In the training and testing cohorts, the preoperative predictive performances of the arterial phase and the delayed phase were the highest, followed by the other two phases. The AUCs of arterial and delayed phases in the training cohorts were respectively 0.822 (95% *CI* 0.674-0.935) and 0.838 (95% *CI* 0.724-0.943) with the SVM model, 0.703 (95% *CI* 0.597-0.855) and 0.711 (95% *CI* 0.627-0.854) with the KNN model, and finally 0.751 (95% *CI* 0.642-0.889) and 0.742 (95% *CI* 0.645-0.857) with the LR model. Based on our study data, **Table 2** demonstrates that the SVM model attained the best performance. The AUCs of ROC curves of three models in the training and testing cohorts are presented in **Figure 4**. The Delong test showed no statistical difference between the arterial phase and the delayed phase, and between the venous phase and the plain scan (*p*=0.471, 0.685), but there were statistical differences between the arterial and delayed phases and the other two phases (*p*=0.017-0.047).

**Table 2:** ROC curve analysis of different machine models in the training and testing cohorts.

Parameter		AUC (95% CI)			Accuracy			Sensitivity			Specificity		
		SVM	KNN	LR	SVM	KNN	LR	SVM	KNN	LR	SVM	KNN	LR
NP	Train	0.787 (0.658-0.899)	0.688 (0.579-0.812)	0.723 (0.607-0.844)	0.701	0.649	0.665	0.694	0.603	0.647	0.832	0.761	0.767
	Test	0.691 (0.618-0.836)	0.612 (0.497-0.748)	0.652 (0.513-0.763)	0.659	0.582	0.601	0.613	0.582	0.654	0.766	0.621	0.585
AP	Train	0.822 (0.674-0.935)	0.703 (0.597-0.855)	0.751 (0.642-0.889)	0.788	0.655	0.736	0.786	0.647	0.736	0.849	0.733	0.797
	Test	0.752 (0.619-0.875)	0.611 (0.452-0.707)	0.623 (0.495-0.758)	0.736	0.601	0.666	0.726	0.609	0.724	0.784	0.676	0.677
VP	Train	0.795 (0.697-0.912)	0.659 (0.531-0.814)	0.677 (0.561-0.794)	0.745	0.647	0.681	0.727	0.618	0.746	0.809	0.734	0.729
	Test	0.719 (0.613-0.817)	0.641 (0.526-0.775)	0.637 (0.513-0.751)	0.692	0.604	0.589	0.644	0.559	0.587	0.731	0.678	0.657
DP	Train	0.838 (0.724-0.943)	0.711 (0.627-0.854)	0.742 (0.645-0.857)	0.783	0.676	0.726	0.752	0.606	0.612	0.834	0.718	0.753
	Test	0.751 (0.647-0.847)	0.631 (0.532-0.762)	0.639 (0.527-0.763)	0.715	0.628	0.638	0.715	0.581	0.607	0.755	0.622	0.662

SVM, support vector machine; KNN, k-nearest neighbors; LR, logistic regression; AUC, area under the curve; CI, confidence interval; NP, Non-enhanced phase; AP, Arterial phase; VP, Venous phase; DP, Delayed phase.

### Clinical Features

**Figure 5:**
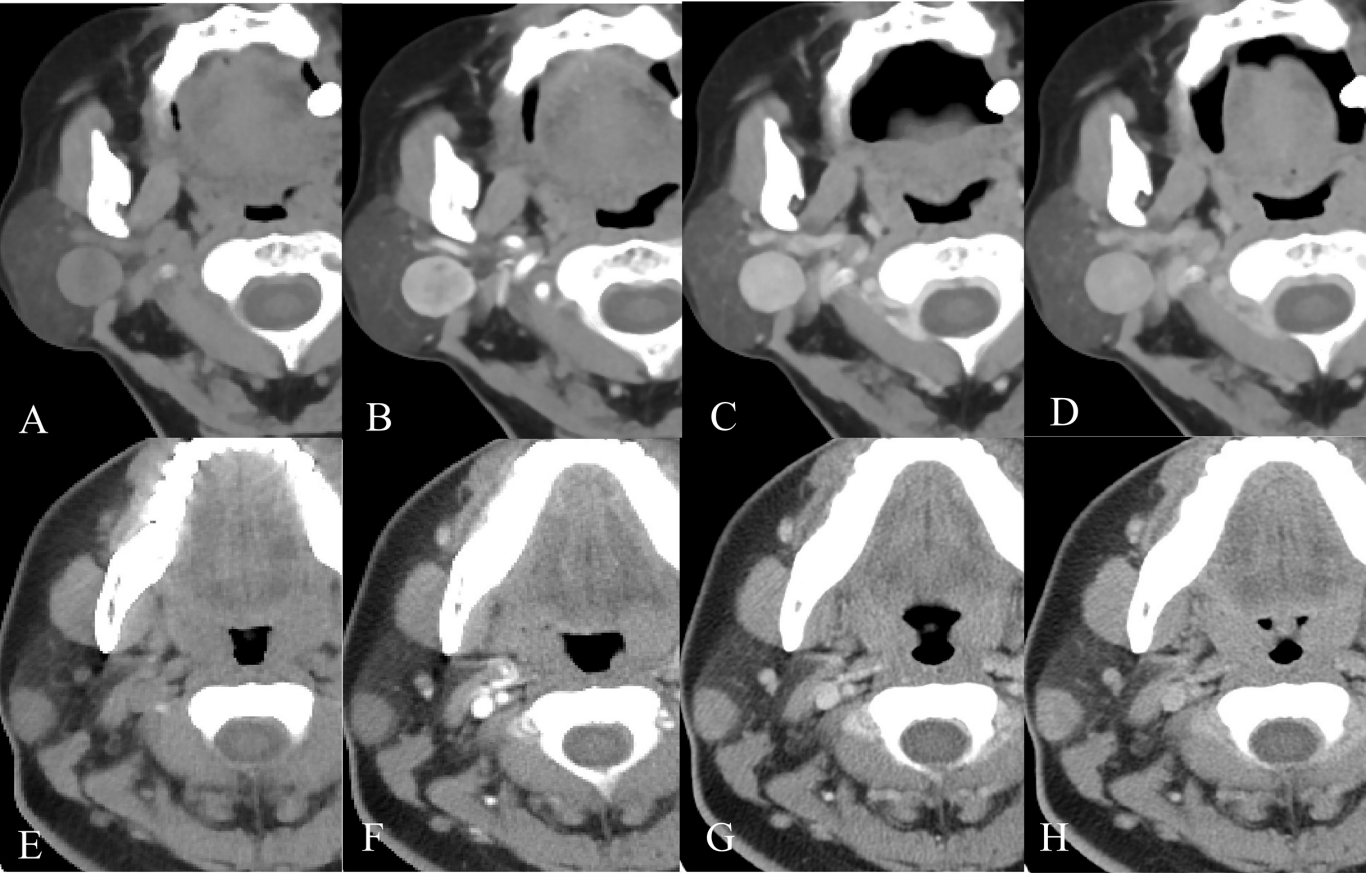
Example images of four-phase CT scans of PA and BCA. **(A-D)** Axial CT images of a 68-year-old female patient with a right-sided painless neck mass in the plain, arterial, venous, and delayed phases, separately. After CT examination, surgical pathology confirmed that the mass was BCA. **(E-H)** Axial CT images of a 23-year-old female patient with a right-sided painless neck mass in the order of plain, arterial, venous, and delayed phases. It was confirmed as PA by surgical pathology.

A total of 173 patients (70 men and 103 women) were recruited for our study, including 121 patients in the training cohort and 52 patients in the testing cohort. **Table 3** provides detailed information on the clinical and radiological characteristics of the patients. **Figure 5** presents a case analysis of the radiological characteristics of PA and BCA, respectively. As shown in **Table 3** and **Table 4**, univariate and multivariate logistic regression analyses indicated that age and enhanced-peak phase were independent predictors that differentiated PA from BCA. These clinical features were employed to construct a clinical model.

**Table 3:** Clinical and radiological characteristics in the training and testing cohorts.

Variables		Training cohort (n = 121)			Testing cohort(n = 52)		
		PA (n = 85)	BCA (n = 36)	P-value	PA (n = 36)	BCA (n = 16)	P-value
Age(years)		45.39 ± 16.89	52.11 ± 10.18	0.008*^a^	44.94 ± 13.91	59.63 ± 14.25	0.001*^a^
Duration(months)		5.40 ± 14.75	15.89 ± 43.32	0.164^a^	14.78 ± 27.81	43.23 ± 90.26	0.234^a^
Max-diameter(cm)		3.09 ± 1.17	2.40 ± 0.95	0.002*^a^	2.58 ± 0.91	2.28 ± 0.82	0.275^a^
Symptoms	With	1(1.2%)	1(2.8%)	0.546^b^	1(2.8%)	1(6.3%)	0.563^b^
	Without	84(98.8%)	35(97.2%)		35(97.2%)	15(93.8%)	
Sex	Female	49(57.6%)	21(58.3%)	0.944	22(61.1%)	11(68.8%)	0.598
	Male	36(42.4%)	15(41.7%)		14(38.9%)	5(31.3%)	
Smoking	Yes	19(22.4%)	9(25.0%)	0.752	10(27.8%)	4(25.0%)	0.834^b^
	No	66(77.6%)	27(75.0%)		26(72.2%)	12(27.8%)	
Drinking	Yes	18(21.2%)	11(30.6%)	0.269	8(22.2%)	3(18.8%)	0.775^b^
	No	67(78.8%)	25(69.4%)		28(77.8%)	13(81.3%)	
Number	Single	84(98.8%)	35(97.2%)	0.546^b^	36(100.0%)	16(100.0%)	–
	Multiple	1(1.2%)	1(2.8%)		0	0	
Distribution	Left-sided	43(50.6%)	20(55.6%)	0.617	12(33.3%)	4(25.0%)	0.187
	Right-	42(49.4%)	16(44.4%)		24(66.7%)	12(75.0%)	
Shape	Round	74(87.1%)	31(86.2%)	0.889^b^	32(88.9%)	14(87.5%)	0.886^b^
	Non-round	11(12.9%)	5(13.9%)		4(11.1%)	2(12.5%)	
Boundary	Clear	82(96.5%)	34(94.4%)	0.618^b^	36(100.0%)	16(100.0%)	–
	Unclear	3(3.5%)	2(5.6%)		0	0	
Location	Superficial	51(60.0%)	25(69.4%)	0.521	27(75.0%)	13(81.3%)	0.617^b^
	Deep	18(21.2%)	7(19.4%)		8(22.2%)	2(12.5%)	
	Both	16(18.8%)	4(11.1%)		1(2.8%)	1(6.3%)	
Density	Solid	63(74.1%)	30(83.3%)	0.305^b^	27(75.0%)	12(75.0%)	1.000^b^
	Cystic	3(3.5%)	2(5.6%)		0	0	
	Mixed	19(22.4%)	4(11.1%)		9(25.0%)	4(25.0%)	
Calcification	With	5(5.9%)	1(2.8%)	0.448^b^	1(2.8%)	1(6.3%)	0.563^b^
	Without	80(94.1%)	35(97.2%)		35(97.2%)	15(93.8%)	
Cystic areas	With	13(15.3%)	9(25.0%)	0.206	5(13.9%)	5(31.3%)	0.143
	Without	72(84.7%)	27(75.0%)		31(86.1%)	11(68.8%)	
Enhanced-peak phase	Arterial	1(1.2%)	11(30.6%)	<0.001*^b^	2(5.6%)	11(68.8%)	<0.001*^b^
	Venous	7(8.2%)	14(38.9%)		5(13.9%)	4(25.0%)	
	Delayed	77(90.6%)	11(30.6%)		29(80.6%)	1(6.3%)	
Enhancement degree	Slight	21(24.7%)	3(8.3%)	0.007*	8(22.2%)	1(6.3%)	0.003*^b^
	Moderate	35(41.2%)	10(27.8%)		14(38.9%)	1(6.3%)	
	Obvious	29(34.1%)	23(63.9%)		14(38.9%)	14(87.5%)	
Enhanced uniformity	Yes	49(57.6%)	23(63.9%)	0.523	21(58.3%)	11(68.8%)	0.476
	No	36(42.4%)	13(36.1%)		15(41.7%)	5(31.3%)	
Enlarged lymph nodes	With	7(8.2%)	2(5.6%)	0.598^b^	1(2.8%)	0	0.388^b^
	Without	78(91.8%)	34(94.4%)		35(97.2%)	16(100.0%)	

*Represents P < 0.05. PA, pleomorphic adenoma; BCA, basal cell adenoma; Numerical data are presented as mean ± standard deviation, Categorical data as numbers (n%); a represented using student t-test, b indicated using likelihood ratio, and others using chi-square test.

**Table 4:** Univariate and Multivariable logistic regression analysis for selecting clinical features of model development.

Variable	Univariate analysis		Multivariate analysis	
	OR (95% *CI*)	P-value	OR (95% *CI*)	P-value
Age(years)	1.043(1.018-1.068)	0.001*	1.034(1.002-1.066)	0.035*
Duration(months)	1.004(0.999-1.009)	0.113		
Max-diameter(cm)	0.549(0.376-0.800)	0.002*	0.617(0.375-1.013)	0.056
Symptom	2.380(0.326-17.369)	0.393		
Sex	0.888(0.456-1.727)	0.725		
Smoking	1.057(0.498-2.247)	0.884		
Drinking	1.346(0.635-2.852)	0.438		
Number	1.167(0.103-13.157)	0.901		
Distribution	0.972(0.506-1.866)	0.933		
Shape	1.099(0.420-2.878)	0.847		
Margin	1.573(0.255-9.706)	0.625		
Location Superficial		0.530		
Deep	0.711(0.303-1.665)	0.431			
Both	0.604(0.207-1.760)	0.355		
Density Solid		0.482		
Cystic	1.429(0.230-8.873)	0.702		
Mixed	0.612(0.257-1.457)	0.267		
Calcification	0.767(0.150-3.930)	0.750		
Cystic areas	2.108(0.956-4.651)	0.065		
Enhanced-peak phase Arterial		<0.001*		<0.001*
Venous	0.205(0.050-0.838)	0.027*	0.200(0.046-0.863)	0.031*
Delayed	0.015(0.004-0.059)	<0.001*	0.013(0.002-0.070)	<0.001*
Enhancement degree Slight		<0.001*		0.144
Moderate	1.628(0.474-5.585)	0.439	4.464(0.874-22.802)	0.072
Obvious	6.238(2.007-19.390)	0.002*	1.601(0.314-8.160)	0.571
Enhanced uniformity	0.727(0.370-1.428)	0.354		
Enlarged lymph nodes	0.565(0.116-2.756)	0.480		

*Represents P < 0.05. OR, Odds ratio; CI, confidence interval.

### Combined Model Construction and Validation

**Figure 6:**
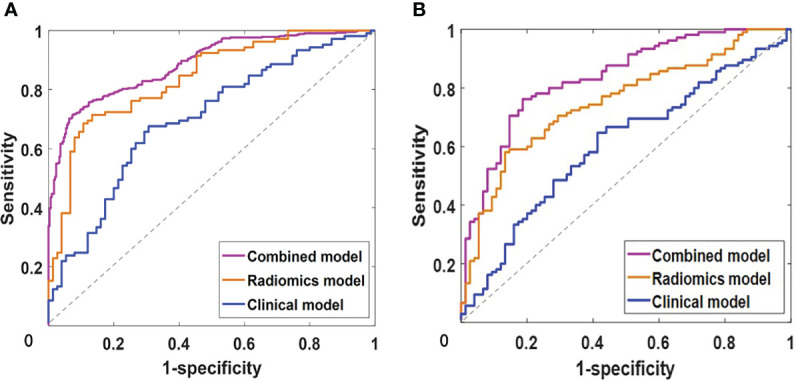
The receiver operating characteristics (ROC) curve analysis of clinical, radiomics and combined models with support vector machine in training **(A)** and testing **(B)** cohorts.

By integrating clinical predictors (age, enhanced-peak phase) with 16 radiomic features, we developed a combined model. **Table 2** and **Table 5** demonstrate that the combined model of the SVM classifier had the best predictive performance through comprehensively comparing the AUCs, specificity, and sensitivity of different models. The combined model had the greatest AUCs (0.885 and 0.834), accuracy (0.847 and 0.813), sensitivity (0.797 and 0.755), and specificity (0.953 and 0.904) in both the training and testing cohorts. **Figure 6** depicts the ROC curves of the different models in the training and testing cohorts.

**Table 5:** ROC curve analysis of the clinical model and the combined model with support vector machine in the training and testing cohorts.

Models		AUC (95% CI)	Accuracy	Sensitivity	Specificity
Combined	Training	0.885 (0.762-0.993)	0.847	0.797	0.953
	Testing	0.834 (0.707-0.954)	0.813	0.755	0.904
Clinical	Training	0.697 (0.524-0.809)	0.597	0.688	0.585
	Testing	0.602 (0.496-0.758)	0.543	0.669	0.543

AUC, area under the curve; CI, confidence interval.

## Discussion

This study provided a detailed analysis of the advantageous scan phases and different radiomic models that differentiate PA and BCA. Our results revealed that the arterial and delayed phases were superior for differentiating parotid PA and BCA, and radiomics might help distinguish PA from BCA. In particular, the combined model, including clinical predictors and radiomic features, showed the best diagnostic performance in this study.

There were numerous parallels between PA and BCA. For instance, both were more common in females, were painless masses, were located in the parotid superficial lobe, were mostly unilateral, round or oval, with clear boundaries, and possible malignant transformations (6, 7, 25). Some studies indicated that the average age of BCA was about 10 years older than PA (6, 26). In our study, PA and BCA patients’ mean age was 45.3 ± 15.9 years and 54.4 ± 11.8 years, which was similar to the results of previous studies. In previous reports (6, 13), the average diameter of BCA was generally smaller than that of PA. This trend was also present in the current study, but there was no statistical difference, which might correlate with the uneven samples. Mukai et al. (25) reported that cystic components were observed more frequently in BCA than in PA, and were comparatively larger in BCA. In this study, 14 of 52 BCAs (26.9%) had cystic components, while 18 of 121 PAs (14.9%) had, although the difference was not statistically significant. Nevertheless, the overlapping features of BCA and PA may make preoperative diagnosis challenging, and there is no consensus to date to distinguish between the two. Therefore, a new, non-invasive, objective method is urgently required to differentiate BCA from PA.

Radiomics is a non-invasive, promising field that provides valuable information on diagnosis, prognosis, and individualized therapy through objective, quantitative assessments based on tumor heterogeneity (27). Our results indicated that several GLCM features were robust among radiomic signatures and participated in constructing predictive models. GLCM features capture comprehensive information about the direction, adjacent interval, and change amplitude of the image gray level, analyze the local patterns of the image and the basis of their arrangement rules; and reflect the relevant intra-tumor heterogeneity (28). This may be related to the tumor pathology types. PA contains mixed components such as mucus, parotid gland, and cartilage-like tissue, while BCA consists of basaloid cells, with a large number of vascular networks (capillaries and veins) arranged along the endothelium (7).

Several studies demonstrated that multiphase CT offered valuable information for tumor characterization, such as enhancement patterns, vascular structures, and tumor relationships to surrounding structures (29, 30). There were four histological subtypes of BCA, including solid, trabecular, tubular, and membranous, of which the solid subtype was the most common, while the membranous subtype had a high recurrence rate and malignant transformation (25, 26). Enhancement patterns vary by BCA histological type, with solid types displaying substantial early enhancement and a washout pattern like WT, while other types exhibit weaker early enhancement and the gradual enhancement pattern observed in PA (31). Consistent with the results of this study, we found that the diagnostic efficacy of differentiating BCA from PA in CT plain and three-phase CT-enhanced scans was best in the arterial and delayed phases. We speculated that it might be related to the different tissue components in PA and BCA.

It is crucial to develop robust predictive models to select valid indicators and appropriate modeling approaches (32). In this study, we investigated three frequently utilized machine learning classifiers, including linear LR, nonlinear KNN, and SVM. KNN primarily relied on the limited adjacent samples around the undetermined samples to determine the samples, which applied to handling the pending sample sets with class domain crossovers or more overlaps, but when the sample numbers were uneven, it would cause biased judgment results (33). With satisfactory robustness and effectiveness, SVM achieved almost the same performance as a large number of training samples with small training samples (34, 35). The combined model integrating clinical and radiomic features, with AUC and accuracy of 0.873 and 0.856 in the training cohort and 0.819 and 0.802 in the test cohort, exhibited the optimal discrimination performance among all predictive models. It suggested that although radiomic features of parotid tumors provide better discrimination than radiological features, clinic-radiological information was also valuable; only when combined with these features could the model accurately assess the entire tumor and facilitate precision medicine.

This study still had several limitations. First, it was a retrospective single-center study with potential selection bias. Second, several controversies connected with subjectivity in delineating the boundaries of manual segmentation (36) required further investigation of semiautomatic or completely automatic methods for tumor segmentation. Third, with an uneven ratio of patients between PA and BCA in this study, it was unclear whether this affected the modeling, and further large-sample, multicenter, and prospective studies would be required.

## Conclusion

The present study demonstrated that the SVM model of the arterial and delayed phases contributed to distinguishing BCA from PA. Significantly, the combined model integrating clinical and radiomic features exhibited the best diagnostic performance. Before actual clinical application, multicenter and prospective studies with larger datasets should be conducted to validate the combined model.

## Data Availability Statement

The raw data supporting the conclusions of this article will be made available by the authors, without undue reservation.

## Ethics Statement

The studies involving human participants were reviewed and approved by The First Affiliated Hospital of Chongqing Medical University. Written informed consent from the participants’ was waived, the legal guardian/next of kin was not required to participate in this study in accordance with the national legislation and the institutional requirements.

## Author Contributions

Study design and conception, Y-LZ, Y-NZ, DZ, and MW. Data collection, Y-LZ, J-NG, X-YZ, and X-YL. Radiomics data analysis, Y-NZ. Data analysis and discussion, Y-LZ, Y-NZ, C-F L, DZ, and MW. Manuscript writing, Y-LZ. Supervision and Revision, DZ and MW. All authors contributed to the article and approved the submitted version.

## Funding

This work was supported by the National Natural Science Foundation of China (81801717 and 82103206) and the Chongqing basic research and frontier exploration project of Chongqing Science & Technology Commission (cstc2021jcyj-msxmX0018). We thank the funders supporting this study.

## Conflict of Interest

The authors declare that the research was conducted in the absence of any commercial or financial relationships that could be construed as a potential conflict of interest.

## Publisher’s Note

All claims expressed in this article are solely those of the authors and do not necessarily represent those of their affiliated organizations, or those of the publisher, the editors and the reviewers. Any product that may be evaluated in this article, or claim that may be made by its manufacturer, is not guaranteed or endorsed by the publisher.
