# Supplementary material for: Comparison of Different Machine Models Based on Multi-Phase Computed Tomography Radiomic Analysis to Differentiate Parotid Basal Cell Adenoma From Pleomorphic Adenoma
Source: Front Oncol. 2022 Jul 12;12:889833. doi: 10.3389/fonc.2022.889833 (PMC9315155; doi:10.3389/fonc.2022.889833)
Supplement: Supplementary file 1 [file DataSheet_1.docx]

Supplementary Material

# Supplementary Data

The definitions of the imaging features are listed as follows:

Max-diameter: The sizes of the tumors were measured by determining the maximal cross-sectional diameter.

Symptoms (with or without): We assessed the symptoms based on the clinical record, including pain/tenderness or facial nerve palsy.

Number: We observed the lesions of parotid tumor patients on the picture archiving and communication systems (PACS) of our hospital. If there was only one lesion, it was considered single, and if there were two or more lesions, it was multiple.

Location: The lobes were divided by the retromandibular vein into shallow and deep lobes. Therefore, the location of the tumor was defined as in deep lobe if it is interior to the retromandibular vein, or in shallow lobe exterior to the retromandibular vein. If the tumor grows across two lobes, it was defined as in both lobes.

Density: Solid density was defined as the CT value of the tumor resembling the soft tissue CT value. Cystic density was defined as the CT value of the tumor with a watery CT value (<20Hu). Mixed density was defined as the CT value of the tumor with both soft tissue and watery CT value.

Calcification: Calcification was defined as the CT value of the foci within the tumor is higher than 100Hu.

Cystic areas: cystic area was defined as having a CT scan attenuation of 20 HU or less.

Enhanced-peak phase: We measured CT values (in HU) on non-enhanced, arterial and venous CT scans by placing the largest possible circular region of interest within the solid portion of the lesion with caution to avoid the cystic area. The phase of the highest CT values was defined as enhanced-peak phase.

Enhancement degree: Obvious enhancement was defined as the CT value of tumor enhancement on postcontrast CT is 40Hu higher than it on non-enhanced CT scan. Slight enhancement was defined as the CT value of tumor enhancement on postcontrast CT below 20 Hu on the basis of non-enhanced CT value. Moderate enhanced CT values fell somewhere in between.

Enlarged lymph nodes (with or without): We evaluated ipsilateral lymph node metastases based on imaging features and intraoperative records. The maximal axial dimension criteria for metastatic lymph nodes on imaging were 8 mm for retropharyngeal nodes and 10 mm for all other node levels.
